# Oxidative Stress in Patients with Alzheimer's Disease: Effect of Extracts of Fermented Papaya Powder

**DOI:** 10.1155/2015/624801

**Published:** 2015-04-07

**Authors:** Mario Barbagallo, Francesco Marotta, Ligia J. Dominguez

**Affiliations:** ^1^Geriatric Unit, Department DIBIMIS, University of Palermo, Via del Vespro, 129, 90127 Palermo, Italy; ^2^UOC di Geriatria e Lungodegenza, AOUP Azienda Universitaria Policlinico, Via del Vespro, 129, 90127 Palermo, Italy; ^3^ReGenera Research Group for Aging Intervention, Milano, Italy

## Abstract

Brain tissue is particularly susceptible to oxidative stress (OS). Increased production of reactive oxygen species (ROS), reduced antioxidant systems, and decreased efficiency in repairing mechanisms have been linked to Alzheimer's disease (AD). Postmortem studies in AD patients' brains have shown oxidative damage markers (i.e., lipid peroxidation, protein oxidative damage, and glycoxidation). Fermented papaya (FPP, a product of* Carica papaya Linn* fermentation with yeast) is a nutraceutical supplement with favorable effects on immunological, hematological, inflammatory, and OS parameters in chronic/degenerative diseases. We studied 40 patients (age 78.2 ± 1.1 years), 28 AD patients, and 12 controls. Urinary 8-OHdG was measured to assess OS. Twenty AD patients were supplemented with FPP (Immunage, 4.5 grams/day) for 6 months, while controls did not receive any treatment. At baseline, 8-OHdG was significantly higher in patients with AD versus controls (13.7 ± 1.61 ng/mL versus 1.6 ± 0.12 ng/mL, *P* < 0.01). In AD patients FPP significantly decreased 8-OHdG (14.1 ± 1.7 ng/mL to 8.45 ± 1.1 ng/mL, *P* < 0.01), with no significant changes in controls. AD is associated with increased OS, and FPP may be helpful to counteract excessive ROS in AD patients.

## 1. Introduction

Alzheimer's disease (AD) is the most common neurodegenerative disorder, and its incidence increases with age [1]. AD is characterized by the presence of several pathological hallmarks including neuronal loss, formation of senile plaques composed by extracellular deposits of amyloid beta (A*β*) caused by an abnormal processing of amyloid-beta precursor protein (APP), intracellular neurofibrillary tangles (NFT) composed of aggregated hyperphosphorylated tau proteins in brain, proliferation of astrocytes, and activation of microglial. These features are accompanied by mitochondrial dysfunction and alterations in neuronal synapses [1]. The molecular and pathophysiological mechanisms that underlie AD still have many dark sides. Even though AD is multifactorial, its etiology and the exact mechanism that triggers the pathological alterations are still not clear. Although most studies have suggested that the A*β* peptide (amyloid cascade hypothesis) may initiate and/or contribute to the pathogenesis of AD, the mechanisms through which it causes neuronal loss, and tau abnormalities still remain poorly understood. Reactive oxygen species (ROS) and reactive nitrogen species (RNS), including superoxide anion radical, hydrogen peroxide, hydroxyl radical, singlet oxygen, alkoxyl radicals, peroxyl radicals, and peroxynitrites, contribute to the pathogenesis of numerous human degenerative diseases [2] and have been implicated in the pathogenesis of neurodegenerative disorders including AD and Parkinson's disease, among others [3]. The production of reactive oxygen species (ROS) seems to be involved in triggering and maintaining the degeneration cycle of AD, causing the damage of mitochondrial DNA and of the electron transport chain, which leads to an increased production of ROS [4]. Brain tissue is particularly susceptible to oxidative damage. The metabolism of brain tissue requires high energy levels and it consumes approximately 20% of the total body oxygen despite the fact that it comprises less than 2% of total body weight. It is very rich in easily oxidizable polyunsaturated fatty acids and transition metal, such as iron and ascorbate, which are key players in oxidation and facilitate the formation of oxygen free radicals. The brain is also characterized by a low content of antioxidant systems [5].

The generation of ROS, which are toxic, is a part of normal metabolism in a biological system. Free radicals are extremely reactive species, which once formed can start a series of reactions that are harmful to the cell. It is important to emphasize that even under normal conditions there is a physiological cellular production of free radicals, which is normally counterbalanced by endogenous enzymatic cellular antioxidants systems. The balance between the production of reactive oxygen species and antioxidants is essential in a biological system to prevent adverse effects of oxidative stress. The damage caused by free radicals is caused by an imbalance between their production and their neutralization by cellular antioxidant systems in the human body [6, 7]. Both systems (production and neutralization) seem to be altered in AD and these changes have been suggested to play a major role in the process of age-related neurodegeneration and cognitive decline [8]. The free radicals thus generated are known to attack macromolecules such as deoxyribonucleic acid, proteins, lipids, and carbohydrates. This leads to either onset or acceleration of degenerative disorders. The main damage occurs for integration with cellular macromolecules essential to survival, such as DNA, proteins, and polyunsaturated fatty acids (which make up the cell membrane) [9]. Thus, ROS have been shown to trigger a variety of damage to cellular DNA and RNA, causing peroxidation of membranes and neuronal damage. In addition, the alterations of oxidative metabolism may render the brain more susceptible to further damage from A*β*, which in turn has a prooxidant action [10]. Accumulating evidence suggests that brain tissues in AD patients are exposed to oxidative stress during the development of the disease [11]. Oxidative stress and the following cellular damage caused by protein oxidation, lipid oxidation, DNA oxidation, and glycoxidation are closely associated with the development of cognitive decline in AD [9, 12].

Because free radicals and oxidative DNA damage may have a central role in age-related diseases such as AD, a protection from oxidative stress, and subsequent DNA damage may represent a basic approach for elongation of healthy age and treatment of such age-related diseases. In vitro antioxidant such as N-acetylcysteine or genetic disruption of the DNA damage response pathway by checkpoint kinase deletion can rescue many deficits and eventually elongates significantly lifespan [13]. These observations further indicate the important role of mitochondria, ROS, and DNA damage in aging and neurodegenerative diseases. In the past, randomized controlled intervention studies in AD, with antioxidants, such as selegiline or vitamin E, have produced modest but significant results [14]. Fermented papaya preparation (FPP), produced by fermentation of* Carica papaya *Linn by using yeast, is a food supplement that possesses beneficial and potent antioxidant properties that may be helpful against age-related and disease-related increase in oxidative stress [15]. FPP exhibits anti-inflammatory, antioxidant, and immunostimulatory action and induction of antioxidant enzymes [16].

In neurological conditions, oral administration of FPP in mice attenuated the reduction of short- and long-term memory induced by scopolamine [17]. Because of the above-described role of free radicals in the pathophysiology of chronic neurodegenerative diseases, it has been suggested a possible role for the antioxidant action of FPP in counteracting the oxidative stress associated with these conditions [15]. Therefore, we conducted the present study aiming to explore the effects of oral FPP on oxidative stress in AD patients.

## 2. Methods

We have measured oxidative stress in patients with initial or mild AD compared to age-matched control patients without AD. We have also tested the ability of FPP to reduce the excessive production of free radicals in patients with AD [12]. Oxidative stress was assessed by means of an enzyme immunoassay for the measurement of 8-hydroxy-2′-deoxyguanosine (8-OHdG) in the urine. Detection of 8-OHdG, a nucleic acid modification predominantly derived from hydroxide attack of guanidine, allows for assessment of more immediate oxidative damage [18]. We studied 40 patients (23 women and 17 men, mean age 78.2 ± 1.1 years) evaluated at the Alzheimer Evaluation Units of the University Hospital of Palermo. Twenty-eight patients were recruited after being diagnosed with early mild AD according to the criteria of the DSM-IV and NINCDS-ADRDA, while the other 12 were control patients of the same age.

The patients were not being treated with any other neurotrophic drug during the whole duration of the study. The 28 AD patients were divided into two groups; participants in group 1 (*n* = 20 patients) were treated for 6 months with a supplement of FPP (known commercially as Immunage, prepared by fermenting the* Carica Papaya *Linn at the Osato Research Institute, Gifu, Japan) at a dose of 4.5 grams per day p.o. in a single dose. Patients of group 2 (8 AD patients) did not receive any treatment.

## 3. Results and Discussion

**Figure 1:**
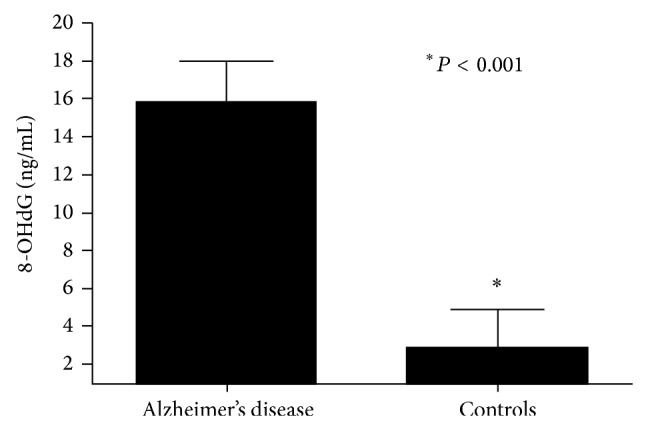
8-Hydroxy-2′-deoxyguanosine (8-OHdG) level in patients with Alzheimer's disease and in controls.

**Figure 2:**
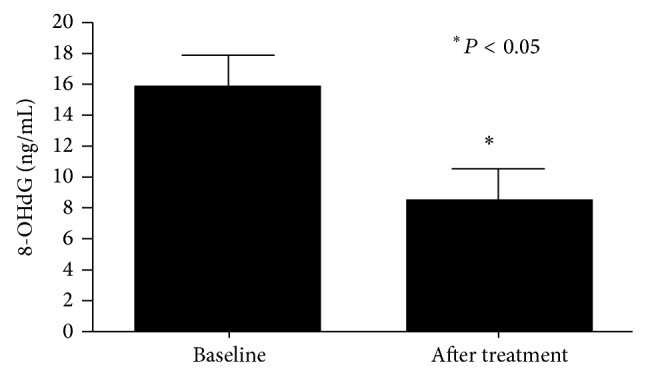
8-Hydroxy-2′-deoxyguanosine (8-OHdG) level in patients with Alzheimer's disease (group 1) before and after fermented papaya powder (FPP) supplementation.

**Figure 3:**
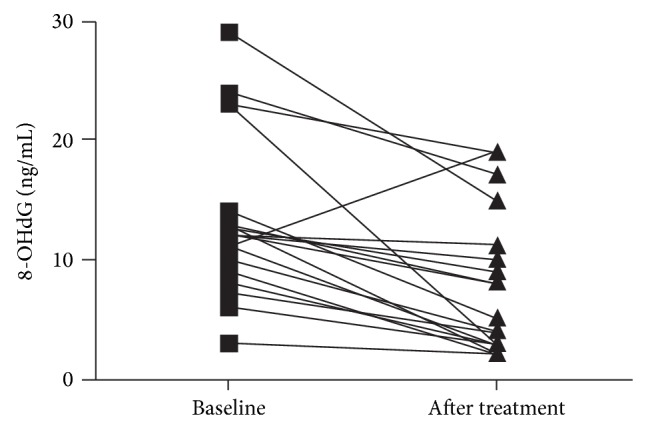
8-Hydroxy-2′-deoxyguanosine (8-OHdG) level in each of the 20 patients with Alzheimer's disease (group 1) before and after fermented papaya powder (FPP) supplementation.

The clinical characteristics of the study participants are shown in Table 1. At baseline, 8-OHdG was significantly higher in patients with AD versus controls (13.7 ± 1.61 ng/mL versus 0.12 ± 1.6 ng/mL, *P* < 0.01, Figure 1). In group 1, supplementation with FPP significantly reduced 8-OHdG levels (from 14.1 ± 1.7 ng/mL to 8.45 ± 1.1 ng/mL, *P* < 0.01, Figure 2), while 8-OHdG did not change significantly in group 2 (not supplemented), showing a nonsignificant trend towards an increase (from 12.5 ± 1.9 ng/mL to 19.6 ± 4.1 ng/mL, *P* = NS). In the 20 patients treated with FPP, oxidative stress as measured by 8-OHdG was reduced in all but one patient (Figure 3). There were no significant changes in clinical MMSE evaluation and/or on any other laboratory parameters examined.

Numerous alterations of oxidative metabolism such as increased production of ROS metabolites and/or a reduction in the efficiency of antioxidant systems and repair capability of damaged molecules are present in AD and have been connected to its onset. Mitochondrial oxidative damage has been found to be excessive in the brains of aged people, especially AD patients and AD-like transgenic animal models. The damage caused by oxidative stress is one of the earliest pathophysiological events in the development of AD; it also seems to precede the formation of amyloid plaques and neurofibrillary tangles. Markers of DNA damage, particularly oxidative DNA damage, have been largely found in brain regions, peripheral tissues, and biological fluids of AD patients. Moreover, there is evidence that oxidative damage is one of the earliest detectable events within the progression from a normal brain to dementia [9, 19]. Almost one decade ago, a decrease in the DNA base excision repair activity was observed in postmortem brain regions of AD individuals, leading to the hypothesis that the brain in AD might be subjected to the double insult of increased DNA damage, as well as deficiencies of DNA repair pathways [20]. Autopsy studies on brain tissue from AD patients' brain tissue from AD patients have confirmed the presence of numerous signs of oxidative stress, such as A*β*-induced oxidative DNA damage and mitochondrial dysfunction, together with an increase in lipid peroxidation, proteins, and glycides oxidation [21], and a reduction of the antioxidant enzyme systems [22]. In vitro studies have shown that the neurotoxic properties of A*β* may be mediated by oxygen radicals. Amyloid deposits are associated with an overexpression of markers of oxidative stress, increased structural abnormalities of mitochondria, and mitochondrial DNA damage [23].

Age is the greatest risk factor for AD. Aging and chronic diseases are themselves associated with an increase in oxidative stress. The concentrations of oxidative-damaged proteins, lipids, and DNA have been reported to increase with age. This increase of oxidative stress during the aging process may contribute in part towards neurodegeneration in AD. The temporal association of the age related increased levels of ROS with the formation of the senile plaque provides further evidence that aging-induced alterations in brain oxidative status may be a major factor in triggering enhanced production and deposition of A*β* in AD [19].

We have previously shown that chronic diseases, such as diabetes mellitus type 2 or cardiovascular conditions, accelerate the age-dependent increase in oxidative stress [24]. A further derangement in oxidative stress balance may be caused by chronic inflammation of aging. Aging is characterized by a chronic, low-grade inflammation, and this phenomenon has been termed as inflammaging [25]. Aging and chronic disease create a cascade of events that can be best characterized as an asymptomatic inflammatory process. This cascade of events is mediated by cytokine interleukins 1 and 6 (IL-1alpha, and IL-6), nitric oxide (NO), and oxidative stress [26]. Inflammation has been suggested to be another responsible factor in AD and is presumed to be mediated through the cross talk among the amyloid, astrocytes, and microglia [27]. These reactions lead to altered neuronal function and the inflammatory injury. Thus, in patients with AD we have recently shown an increase of oxidative stress [12] and an alteration of the immunoinflammatory responses, with an increased cytokine production, that may have a potential causal role in contributing to an augmented oxidative stress [28]. Several studies have shown that A*β* may produce an increase in oxidative stress via several mechanisms, either increase in ROS production, decrease in the enzyme activities involved in the antioxidant defense system, or altering mitochondria function. Nerve cell insults caused by A*β* brain deposition may itself induce oxidative changes [29], and metals concentrated in amyloid deposits, such as copper, may as well contribute to the oxidative insults observed in AD-affected brains. Several studies suggest that A*β* increases oxidative stress by increasing lipid peroxidation measured by increased levels of thiobarbituric acid-reactive substances in brain [29]. In addition to lipids, it has been suggested that ROS-mediated reactions with proteins lead to oxidative damage of proteins and DNA in the brain tissue [30]. The accumulation of oxidative stress metabolites present in old age may itself cause an increase susceptibility of the brain to damage from neurotoxic peptides such as soluble or fibrillar A*β*. As the accumulation of A*β* can in turn cause a further production of ROS, it is still unclear whether the excess of oxidative stress is a primary or secondary event in AD. Although there are accumulating evidences suggesting that oxidative stress may be an early event in the onset of AD, this aspect seems to be of relative importance, as the production of ROS, even if secondary, is in turn detrimental to the brain tissue and can further contribute to neuronal damage [31]. This suggests that any effort to the removal and/or prevention of ROS formation may be useful in people with AD [29]. For example, when A*β* has started to aggregate and deposit in the brain, this protein elicits a neuroinflammatory response via the activation of microglia and astrocytes [32, 33]. Following the initial neuroinflammatory response, the neurotoxic by-products of inflammation cause additional oxidative damage to cells. Similarly, the hyperphosphorylated tau fibrils create cytoskeletal stresses and promote neuronal dysfunction [34].

Oxidative stress-induced cell damage and inflammation are implicated in a variety of age-related diseases other than neurodegenerative disorders (such as cancers, diabetes, arthritis, and cardiovascular dysfunctions) and aging. All these conditions could potentially benefit from functional nutraceutical/food antioxidant supplements. Antioxidant defenses may potentially protect the body from the detrimental effects of free radicals. Physiological antioxidants in food such as fruits and vegetables provide a reasonable amount of antioxidants. Although existing knowledge is not definitive, there is rational basis to suggest that antioxidant supplementation and food plant extracts may help protect against a number of neurological diseases in which oxidative stress is implicated and may have a role in the prevention and treatment of age-related disease.

It has been suggested that substances with antioxidant properties or that enhance the endogenous defense system against free radicals may have a role in preventing the onset or in slowing the progression of AD [35]. Dietary antioxidants contribute to increased levels of cellular antioxidant ability, thus decreasing the toxicity of ROS.

FPP has been shown to reduce apoptosis related to oxidative stress and activation of inflammatory cytokines [36, 37] and to contrast the DNA damage related to the production of free radicals induced by several prooxidant substances, including iron ions, copper, benzopyrene, methylguanidine, and aluminum, among others [15, 38–40].

In particular, it has been shown that FPP exerts several protective actions; it inhibits lipid peroxidation [41]; it enhances enzyme antioxidant activities such as glutathione S-transferase in hepatocytes [36] and showed remarkable hepatoprotective activity [42]. In vitro in a cellular model of AD, FPP has neuroprotective activity against *β*-amyloid-mediated copper neurotoxicity in *β*-amyloid precursor protein in a cell culture system [36]. FPP has shown protective properties against iron-mediated oxidative damage to DNA and proteins [43]. In the brain tissue, FPP has been shown to have a neuroprotective effect from oxidative damage, from lipid peroxide level, and superoxide dismutase activity in iron-induced epileptic foci of rats [41]. At the neuronal level, FPP has been shown to have a neuroprotective action, to improve the oxidative status in human neuronal cells and to protect from insults by oxidative stress linked, for example, to the cytotoxicity by aluminum in neuronal cells [44]. Neuroprotective potential evaluated in an AD cell model showed that the toxicity of the A*β* can be significantly modulated and/or reduced by FPP. In vitro FPP has been shown to protect cells by the oxidative damage related to the deposition of A*β*. Treatment with FPP increased the survival of neuronal cells, preventing apoptosis, and was able to decrease the production of hydroxyl radicals and superoxide anion in the cells, as well as decreasing nitric oxide accumulation and intracellular calcium ion [36]. Noda et al., using an ESR technique, confirmed the potent antioxidant inhibitory effect of the FPP, by demonstrating its hydroxyl radical scavenging activity, its superoxide anion radical scavenging activity (SOD-like activity), and its inhibitory effect on hydroxyl radical generation from methylguanidine [40]. At the clinical level, studies in chronic and degenerative disease conditions (i.e., thalassemia, cirrhosis, and diabetes) showed that FPP favorably modulates immunological, hematological, inflammatory, vascular, and oxidative stress damage parameters [15].

Our results suggest that FPP has antioxidant actions in AD patients and that it may be prophylactic food against the age-related and neurological diseases associated with free radical overproduction. Our data confirm that AD is associated with an increased oxidative stress and that the FPP can be useful in helping to counteract the excessive production of free radicals present in patients with AD [12]. The previous in vitro studies are promising and proven preventive action on the damage from A*β* suggesting that it would also be useful to evaluate the action of FPP in the more advanced stages of the disease and in combination with neurotrophic drugs. It would also be interesting to identify the component of FPP neurotrophic action.

In conclusion, dietary factors can modulate physiological functions (including brain function) thereby increasing the economic productivity of a population as a function of health. A greater understanding of the molecular mechanisms of neuroprotection, oxidative stress, and immune function will facilitate definition of the prophylactic potentials of diet, nutritional/food supplements, medicinal plants, and herbal extracts. Although the role of oxidative stress in aging and neurodegenerative and other related diseases is largely accepted, the value of antioxidant strategies is still debatable. This becomes more important when, apart from foods or reasonable lifestyle changes, antioxidant supplements are considered. Well-defined long-term trials are still needed to assess the efficacy of antioxidant strategies or of antioxidant-rich nutritional intervention. Future studies of longer duration and with a larger number of subjects would be useful to assess the potential clinical actions of FPP and the possible relevance to the reduction of oxidative stress on the natural history of the disease.

## Figures and Tables

**Table 1:** Clinical characteristics of study patients.

	AD (group 1) baseline before FPP supplementation	AD (group 2) not supplemented	Controls without AD	
Age (years)	78.1 ± 1.1	78.3 ± 1.0	77.9 ± 1.2	NS
8-OHdG (ng/mL)	14.1 ± 1.7	12.5 ± 1.9	1.6 ± 0.12	<0.001
SBP (mmHg)	132.9 ± 1.9	130.7 ± 2.1	131.0 ± 2.3	NS
DBP (mmHg)	78.6 ± 1.1	77.7 ± 1.2	77.9 ± 1.2	NS
CHOL (mg/dL)	207.9 ± 39	205.8 ± 38	195.7 ± 41	NS
TG (mg/dL)	127.5 ± 47	118 ± 57	112 ± 49	NS
HDL (mg/dL)	43.8 ± 12	47.9 ± 14	47.6 ± 13	NS
LDL (mg/dL)	136.8 ± 35	128.9 ± 40	127.7 ± 41	NS
BMI	24.9 ± 5.5	24.8 ± 6.4	24.1 ± 6.1	NS
MMSE	22.1 ± 1.5	21.9 ± 1.4	28.8 ± 2.1	*P* < 0.01
